# The Treatment of Carbuncles

**Published:** 1899-06

**Authors:** Milton P. Creel

**Affiliations:** Central City, Ky.; President Muhlenberg County Medical Society: President Muhlenberg County Board of Health; Member Kentucky State Medical Society; Member American Medical Association


### Abstracts and Selections.

### The Treatment of Carbuncles.

BY MILTON P. CREEL, M. D., CENTRAL CITY, KY.
President Muhlenberg County Medical Society: President Muhlenberg County Board of Health: Member Kentucky State Medical Society; Member American Medical Association.

There is no affection falling to the lot of human suffering that is attended with more pain and suffering than carbuncles. Besides the pain which they carry in their train, they are attended with much danger. As a cause of death, upon investigation, we will find that the mortality incident upon this affection is by no means contemptible. In this article I shall not deal with the symptoms or pathology of this affection, that being easily obtained by reference to the standard text-books on surgery.

One of the first considerations in the treatment of a patient with carbuncles is to see that he is well and thoroughly nourished. The importance of this is very manifest when we reflect how much debility is associated with the unfolding of a carbuncle. We should give regularly food of a nourishing character, and we must be satisfied that our patient gets enough to sustain his strength. Liquid diet and easily digested solid foods are to be given as regularly as we do our drugs. Milk, predigested foods, and everything which offers no resistance along the line of nourishment will be called into requisition by the wise physician. In this connection I must not omit to mention the value of stimulants in some cases. In patients who are extremely weak, either from the disease itself or from a poorly nourished state of the system existing before the supervention of the carbuncles, it is of the greatest importance to give some stimulant regularly. Whiskey serves us well, but I generally allow the patient to select his own favorite liquor. I give stimulants often enough to keep the volume of the pulse good. There is no rule better than the one Jurgensen lays down; this, he says, "is the rule of consistency." He explains this by saying that stimulants should be given to produce the effect we desire. We must not stand on quantity or dosage, effect on the pulse is what we must obtain; if large doses are requisite and frequent dosage is necessary, we must bring both to bear.

The old writers on surgery and practice advocated the abstraction of blood and the employment of drastic purgatives. It is not worth serious argument to convince the practitioner of the present day that such practice tends to intensify all the serious factors in the case.

I shall now speak of the treatment of carbuncles by drugs and by surgical means. Let me consider the treatment under two heads; First, the internal treatment; second, the treatment by local applications and surgical procedures. By the internal remedies are meant not, of course, foods and stimulants as have already been mentioned, but pure medication to correct the blood dyscrasia which gave rise to the carbuncular conditions. Iodides and sulphide of calcium have been administered, but they are not now relied upon by the profession. Both of these agents have utterly failed to modify in any way the progress of a carbuncle, and they have been tried thoroughly. Iron has also been tried, and it, too, has failed, and is not now relied upon by the profession. For some months I have relied upon ecthol as an internal medicine. I have notes on fifteen cases treated by this agent. I employ it in doses of a teaspoonful every two hours. Its internal administration is the only drug which I can say has ever seemed to abbreviate the carbuncle. It is a corrector of blood dyscrasia, and in the best sense an anti-purulent. In this connection we may say that an anti-purulent is just what our therapeutics has lacked, and it is the first need of the practitioner when he has a carbuncle under his charge. Ordinarily I give no other internal remedy than ecthol. This remedy I continue until the patient has been discharged. But as improvement becomes marked and steadfast, I allow the interval between the doses to grow longer. First, he is given the remedy every two hours, then every four as he gets along substantially well. This given in doses of a teaspoonful acts very promptly in giving, as it were, a check to tissue disintegration. Of course, opiates are often called for to overcome the pain present, in some cases to an almost insufferable extent. Papine is the best way to exhibit this agent, since it does not produce interference with the secretions as in the case with other opiates. I give it in doses of a teaspoonful every one or two hours until the patient has obtained relief from pain.

Coming now to the measures which should be employed locally and surgically, let me say that this part of the treatment is as important as the giving of internal remedies. During the time the inflammation is beginning and up to the time when there is sufficient pus in the pointing carbuncle to justify an incision, I employ flaxseed poultices. These soothe and hasten the formation of the pus. An incision is now made, and the pus all emptied; the cavity is scraped and all the dead inflamed tissue is removed. It is then carefully cleaned with peroxide of hydrogen. Then absorbent cotton saturated with ecthol is applied to the exposed and adjacent surfaces. This is to be reapplied every four or eight hours, as the case in hand seems to warrant. Each opening is to be treated in this manner, and when we see a case of carbuncle with several centers ready to open we should remove as much of the diseased tissue as possible. Great freedom in the employment of the knife often greatly aids us in bringing about a speedy termination of the case in hand. It is the best thing we can do for our patient to lay the carbuncle open and remove all the diseased tissue, and treat the lesion then with ecthol locally. If we employ this agent as our internal remedy, and use it also as a local application, we shall find that our treatment will prove more effective than by methods employed formerly.

I have treated fifteen cases of carbuncles in the manner here outlined, and the duration in each case has been greatly shortened, and the patients naturally got up with less weakness than they otherwise would.

*Before employing this agent, a carbuncle meant a long spell and death or long-continued convalescence. The average duration of my cases under this treatment has been ten days.*

I now give a brief account of several cases treated by the method I have here advocated:

S. C. T., aged 37, a miner by occupation. He had been a sufferer from malarial fever for a month or so, but was able to work. He had a carbuncle about the size of the palm of the hand on the neck. There was a great deal of pain, and fever of 101° F. was present. His carbuncle had five heads or points, and seemed to invite incision, they showing the presence of pus. This was thoroughly opened and the diseased tissue was removed as thoroughly as possible. Peroxide of hydrogen was used to clean out the diseased cavity well, and then absorbent cotton saturated with ecthol was applied constantly throughout the course of the disease. Ecthol in doses of a teaspoonful was given every two hours. This patient began to improve at once, and there was no retrogression. The carbuncle began to take on a healthy action, and this patient was discharged nine days later.

Mrs. B. K. Y., aged 47, had a carbuncle on her face. This was attended with high fever and delirium. This carbuncle had three openings. It was treated as in the former case as regards the local and surgical means employed. Besides these she had to take predigested milk and considerable quantities of wine, so weak was she. She took ecthol internally also, in doses of a teaspoonful every two hours.

J. C. P., aged 55, had a carbuncle on the nape of the neck. He had been a sufferer for years with asthma, and was in a low state of health. This patient I regarded as one who would give me serious trouble, and who would in all probability die. The carbuncle was freely opened and treated in the same way as the first case here recorded as regards the surgical and local measures. He was from the first given predigested foods and stimulants, and ecthol was the only internal medicine he received except some papine to relieve the pain. This man went along slowly, but he recovered fully, and was able to go about his work seventeen days from the time I first saw him.

These cases are selected because they are ones which would test the efficacy of a treatment.—*The Cincinnati Lancet-Clinic,* April 29, 1899.

